# Check-rein technique for Achilles tendon elongation following conservative management for acute Achilles tendon ruptures: a two-year prospective clinical study

**DOI:** 10.1186/s13018-021-02830-7

**Published:** 2021-11-24

**Authors:** Nicola Maffulli, Francesco Oliva, Filippo Migliorini

**Affiliations:** 1 Department of Medicine, Surgery and Dentistry, University of Salerno, Via S. Allende, 84081 Baronissi, SA Italy; 2 School of Pharmacy and Bioengineering, Keele University School of Medicine, Thornburrow Drive, Stoke on Trent, England; 3 Queen Mary University of London, Barts and the London School of Medicine and Dentistry, Centre for Sports and Exercise Medicine, Mile End Hospital, 275 Bancroft Road, London, E1 4DG England; 4 Department of Orthopaedic, Trauma, and Reconstructive Surgery, RWTH Aachen University Hospital, Pauwelsstraße 30, 52074 Aachen, Germany

**Keywords:** Achilles tendon, Achilles rupture, Tendon elongation, Check-rein procedure

## Abstract

**Background:**

Following conservative management for acute Achilles tendon (AT) ruptures, the tendon may heal in continuity, and some patients may present with an elongated Achilles tendon–gastrosoleus complex. This study investigated the efficacy and feasibility of a novel minimally invasive technique, which we named “check-rein procedure”, in patients with intact and elongated AT following conservative management for AT ruptures.

**Methods:**

All patients who underwent the check-rein procedure for elongation of the gastrosoleus–AT complex by one experienced surgeon were prospectively enrolled. The AT resting angle (ATRA) and AT rupture score (ATRS) were assessed at baseline and repeated at 2-year follow-up, as were calf circumference and isometric plantarflexion strength of both ankles.

**Results:**

Forty-three patients (43 procedures) were analysed. The mean time elapsed from injury to surgery was 28.7 ± 7.9 weeks. The mean age at surgery was 38.5 ± 5.7 years. At the last follow-up, ATRS, ATRA, isometric strength difference, and calf circumference of the affected side were increased (*P* < 0.0001). The rate of the return to sport was 98% (42 of 43). No wound complications or rupture were experienced by any patient.

**Conclusion:**

The check-rein technique for AT elongation after conservative management of AT tears is effective and feasible to restore tendon length and calf function. The surgical outcome was influenced by the preoperative performance status, and longer time elapsed from injury to surgery worsens the outcomes.

## Introduction

Acute Achilles tendon (AT) tears are common. Even when managed optimally these injuries result in impaired muscle strength and endurance and may lead to major functional impairment and retirement from sport [1, 2]. There are no clear guidelines for the management of AT ruptures, and treatment is often based on surgeons and patient preferences [2–5]. Currently, a growing trend to manage acute AT rupture conservatively has been reported [1, 6–8]. In terms of functional outcome and re-ruptures, previous randomized controlled trials demonstrated similarity between conservative and surgical management [9, 10]. However, in some patients, though the tendon has healed in continuity and no re-rupture has taken place, conservative management for acute midportion AT ruptures may result in delayed return to sport, persistent sensation of insecurity and instability, and tendon elongation [11–16]. The latter has been estimated to be up to 3% [17–20]. Tendon elongation correlates significantly with clinical outcome, being an important cause of morbidity as it may produce permanent functional impairment [19, 21–23]. To restore effective push off, the AT must be of the appropriate length in relation to the gastrosoleus complex. The management of elongated AT is challenging with limited options and unpredictable results, and reconstruction and augmentation surgery are more technically demanding than primary repair procedures [24, 25].

For these patients with an elongated Achilles tendon healed in continuity, we undertake a minimally invasive AT augmentation using a free semitendinosus tendon autograft, the check-rein procedure, building on our experience matured with the same graft and surgical technique when needing to restore continuity following insertional acute Achilles tendon tears and chronic Achilles tears with large gaps [26, 27]. We hypothesized that this procedure can be effective in patients with elongated gastrosoleus–Achilles tendon complex to restore tendon length and function. This study investigated the efficacy and feasibility of the check-rein procedure in patients with elongated AT following conservative management for AT ruptures. The present investigation evaluated isometric muscle strength, calf circumference, tendon elongation, functional outcome, and return to physical activities following the check-rein procedure.

## Material and methods

### Study design

The present study was conducted according to the Consolidated Standards of Reporting Trials: the CONSORT statement [28]. The present study was conducted in accordance with principles expressed in the Declaration of Helsinki and approved by the Ethical Committee of the University of Salerno (CESa 01252009/Rev2). Informed consent was obtained from all subjects and/or their legal guardian(s). Patients were enrolled at the Department of Orthopaedics of the University School of Medicine and Surgery of Salerno. All patients who underwent the check-rein procedure AT elongation between 2013 and 2019 were considered. The inclusion criteria were: (1) > 3 months conservative management, (2) AT healed in continuity without gap, (3) age 18 to 50 years, (4) unable to stand on tiptoes with the knee extended. The exclusion criteria were: (1) previous AT surgery, (2) uncontrolled chronic disease, (3) infections, (4) pregnancy or lactation, (5) chronic AT tears with tendon gap, (6) previous ACL surgery using hamstring tendon, (7) immunodeficiency, (8) lower limb deformities, (9) acute AT rupture.

### Surgical procedure

All procedures were performed by one experienced surgeon (NM) in a minimally invasive fashion [26, 27]. Low molecular weight heparin thromboprophylaxis was recommended for 6 weeks. Briefly, patients were positioned prone with their feet leaning over the end of the operating table. After leg exsanguination with an Esmarch bandage, a thigh tourniquet was inflated to 300 mmHg. The most proximal portion of the AT and the superior posterolateral corner of the calcaneum were identified and marked. The semitendinosus tendon was harvested from the medial aspect of the popliteal fossa through a 2.5–3-cm transverse incision [29], and its ends prepared with No. 1 Vicryl (Ethicon, Edinburgh, Scotland) whip stitches. A 3-cm longitudinal incision was performed 8–10 cm cranially to the posterosuperior corner of the calcaneus, medial to the midline of the proximal portion of the AT. The proximal Achilles tendon was exposed through the wound. The semitendinosus tendon autograft was then passed transversely in the AT by blunt dissection and secured with resorbable suture to the AT. A 2.5-cm longitudinal incision was made 2 cm distal and lateral to the lateral margin of the palpable distal AT, remaining close to the lateral margin of the AT, preventing damage to the sural nerve. The Kager’s space and the posterosuperior corner of the calcaneum are exposed. A limited oblique osteotomy of the calcaneus, as close as possible to the insertion of the Achilles tendon, was performed using an oscillating saw to produce space to receive the semitendinosus tendon autograft. The calcaneus was drilled at about 45° inclination to the horizontal plane from dorsal to plantar with a Beath pin, and subsequently a 7-mm cannulated headed reamer was used to produce a suitable tunnel. The two ends of the semitendinosus tendon were passed anterior to the AT, retrieved distally through the distal wound, and shuttled in the bone tunnel. With the ankle in maximal plantar flexion, the graft was tensioned distally and fixed in the calcaneal tunnel using an interference screw. The neotendon was then sutured to the distal portion of the Achilles tendon. The wounds were irrigated and sutured in a standard fashion. A below knee weight-bearing synthetic cast was applied with the foot in maximal plantar flexion.

### Post-operative management

From post-operative day 1 (POD1), active flexion and extension of the hallux, toes, and knee were started, along with isometric exercises of calf muscles and toes, and straight leg raises. With the use of elbow crutches, patients were allowed to weight bear on the metatarsal heads as tolerated for short walks (5 min/h) for the first 3 post-operative weeks. After 3 weeks, the cast was removed, a five-heel wedge Aircast boot (XP Walker, DJO, Guildford, UK) was applied, and patients were allowed to fully weight bear with the boot in situ. Ankle exercises started to increase proprioception, plantar flexion, inversion and eversion, while dorsiflexion and stretching remain forbidden. At 4 weeks, patients removed one heel wedge from the brace and were allowed to start eccentric exercises of the gastrosoleus complex. Active plantarflexion, inversion, and eversion exercises against progressively greater resistance began at this stage. From 8 to 10 weeks, patients gradually discontinued the use of the brace. When the patients were not wearing the brace, they were bearing weight on the operated leg and were instructed to use a 15-mm heel wedge. At full removal of the brace, patients were instructed to use the 15-mm heel wedge for another month. Plyometric exercises were allowed at 5 months post-operatively, and patients were allowed to return to their normal activity.

### Study protocol

At admission, patient demographic information was collected: age, sex, time elapsed from injury to surgery. The AT resting angle (ATRA) [30] was measured to evaluate tendon elongation. With the patient prone and the knee flexed to 90°, the ATRA is the angle between the long axis of the fibula and the line from the tip of the fibula to the head of the fifth metatarsal [31]. The AT rupture score (ATRS) was administered to assess functional outcome [32]. Calf circumference was measured with the knee flexed at 90° while seated and the leg hanging over the side of an examination couch. Attention was made to not compress the calf during the measurements. The calf circumference was measured 15 cm below the medial knee joint line using a standard metallic tape measures with 1-mm increments. This process was repeated twice and the average used for analysis. The isometric plantarflexion strength of the gastrosoleus muscle complex was also evaluated at neutral position (0°) as previously described [33]. All these measurements were taken in the operated limb and compared to the healthy contralateral side. Two years post-operatively, patients were invited for final post-operative assessment. The ATRA, ATRS, calf circumference, and isometric plantarflexion strength were evaluated. Moreover, the single leg heel raise test was administered. During this test, patient stood and leaned against the wall to maintain constant balance. They were invited to lift one foot and stand with their weight on the operated foot with knee straight. The test consists in lift up on the toes, hold for 5 s, lower back onto the heel. Patients should repeat this movement as many times as possible. The number of lifts was recorded.

### Statistical analysis

The statistical analyses were conducted by one author (**). The IBM SPSS Software version 25 was used. For descriptive statistics, mean and standard deviation were used. For continuous data, the mean difference effect measure was used. The *t*-test was used to assess significance, with *P* values < 0.05 considered statistically significant. A multivariate analysis was performed to investigate whether patient characteristics at baseline have association with the outcome at the last follow-up. For the multivariate analyses, the STATA/MP 16.1 (StataCorp, College Station, TX) was used, with a multiple linear model regression diagnostic. The test of overall significance was performed through the Chi-square test, with values of *P* < 0.05 considered statistically significant. For pairwise correlation, the Pearson product–moment correlation coefficient (*r*) was used. The final effect was evaluated according to the Cauchy–Schwarz inequality: + 1 (positive linear correlation) and − 1 (negative linear correlation). Values of 0.1 <|*r*|< 0.3, 0.3 <|*r*|< 0.5, and |*r*|> 0.5 were considered to have small, medium, and strong correlation, respectively.

## Results

### Patient recruitment

**Fig. 1:**
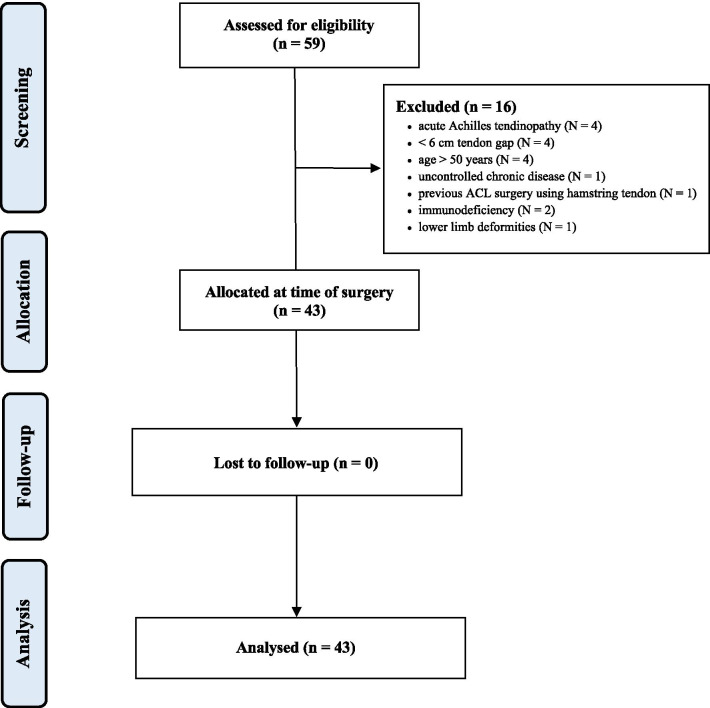
The flow diagram of the recruitment process

A total of 59 patients were assessed for eligibility. A total of 16 patients were excluded because they did not match the eligibility criteria: acute AT re-rupture (*N* = 4), chronic tears with tendon gap (*N* = 4), age > 50 years (*N* = 4), uncontrolled chronic disease (*N* = 1), previous ACL surgery using hamstring tendon(s) (*N* = 1), immunodeficiency (*N* = 2), lower limb deformities (*N* = 1). A total of 43 patients received the planned surgical intervention. No patients were lost at follow-up (Fig. 1).

### Patient demographic

Forty-three patients (43 procedures) were analysed. Seventy-three percentage (33/43) of patients were men, and 51% (22/46) of procedures involved the right side. The mean time elapsed from injury to surgery was 28.7 ± 7.9 weeks. The mean age at admission 38.5 ± 5.7 years. Patient demographics are shown in Table 1.

**Table 1:** Patient demographics at admission

Endpoint	Value
Patients	43
Female	23% (10 of 43)
Male	77% (33 of 43)
Mean age	38.5 ± 5.7
Mean time injury to surgery (weeks)	28.7 ± 9.0
Side right	51% (22 of 43)
Side left	56% (24 of 43)

### Outcomes of interest

At two-year follow-up, the ATRS, ATRA, the isometric strength difference, and the calf circumference of the affected side were all significantly improved (*P* < 0.0001) (Table 2).

**Table 2:** Main results at minimum two years follow-up

Endpoint	Admission	Last follow-up	MD	*P*
ATRS	46.0 ± 5.2	86.6 ± 4.3	40.6	< 0.0001
ATRA	− 4.7 ± 1.8	− 0.9 ± 0.8	3.8	< 0.0001
Isometric strength	211.7 ± 38.3	365.6 ± 31.4	153.9	< 0.0001
Calf circumference	355.3 ± 46.6	342.9 ± 45.3	7.5	< 0.0001

MD, mean difference

Although the calf circumference and the isometric strength were improved, the difference with the contralateral side remained significant at the last follow-up (*P* = 0.0001) (Table 3).

**Table 3:** Isometric strength and calf circumference between operated and contralateral sides

Endpoint	Operated side	Contralateral side	MD	*P*
Isometric strength difference at baseline	211.7 ± 38.3	410.1 ± 29.2	198.41	< 0.0001
Isometric strength difference at follow-up	365.6 ± 31.4	411.1 ± 29.1	45.49	< 0.0002
Calf circumference difference at baseline	355.3 ± 46.6	383.2 ± 46.4	27.88	< 0.0003
Calf circumference difference at follow-up	342.9 ± 45.9	362.9 ± 45.3	20.00	< 0.0004

At two-year follow-up, the average single leg heel raise test was 40.4 (23 to 55). By the last follow-up, the rate of the return to physical activity was 98% (42 of 43). Twenty-six percentage (11 of 43) of patients reduced their activity level, 19% (8 of 43) returned to their preoperative level, and 53% (23 of 43) declared to have improved their activity level since the time of surgery. No complications were experienced by any patient.

### Multivariate analyses

A longer time from injury to surgery was associated with higher ATRA at the last follow-up (*r* = 0.4; *P* = 0.008) and lower functional assessment (*r* = -0.4; *P* = 0.009). ATRA at admission was positively associated with the ATRA at the last follow-up (*r* = 0.4; *P* = 0.009). Isometric strength at admission was positively associated with isometric strength (*r* = 0.6; *P* = 0.0001) and functional assessment (*r* = 0.6; *P* = 0.0003) at the last follow-up. Greater calf circumference at admission was strongly and positively associated with a greater calf circumference at the last follow-up (*r* = -0.9; *P* = 0.0001). No further statistically significant associations were found. The results of the multivariate analysis are shown in greater detail in Table 4.

**Table 4:** Results of the multivariate analysis

Endpoint	ATRS at last FU		ATRA at last FU		Isometric strength at last FU		Calf circumference at last FU		Functional assessment at last FU	
	*r*	*P*	*r*	*P*	*r*	*P*	*r*	*P*	*r*	*P*
Time injury to surgery (*weeks*)	0.05	0.7	0.39	0.008	0.14	0.3	− 0.05	0.7	− 0.40	0.009
Sex	− 0.17	0.2	0.03	0.8	0.07	0.6	− 0.11	0.4	− 0.35	0.2
Age at admission	0.12	0.4	− 0.05	0.7	− 0.01	0.9	0.38	0.1	− 0.13	0.4
ATRS at admission	0.19	0.2	0.04	0.7	0.04	0.7	0.12	0.4	− 0.01	0.9
ATRA at admission	− 0.09	0.5	0.39	0.009	− 0.14	0.3	0.20	0.1	0.02	0.9
Isometric strength at admission	0.04	0.7	0.04	0.8	0.61	0.0001	− 0.10	0.5	0.58	0.0003
Calf circumference at admission	− 0.19	0.2	0.26	0.08	− 0.08	0.5	0.94	0.0001	− 0.06	0.6

## Discussion

The management of acute AT rupture is debated [2–5]. Given the comparable results of conservative and surgical management reported by previous randomized clinical trials [9, 10], the trend to manage these patients conservatively is increasing [1, 6–8]. Although conservative management allows the tendon to heal, the AT may elongate and impair the functional ability of the gastrosoleus–AT complex [11–16]. The present study demonstrated that the check-rein procedure for elongation of the AT following conservative management of AT rupture is effective and feasible to restore tendon length and calf function. Calf circumference and isometric strength were increased at two years post-operatively, but they were still significantly decreased compared to the contralateral side. The outcome was influenced by the preoperative status of the patients, and longer time elapsed from injury to surgery negatively impacts the outcomes.

Rupture of the AT produces hypotrophy of the gastrosoleo complex [34]. In this study, although improved, calf circumference did not reach the values of the contralateral side at the last follow-up. Muscle weakness can persist in the long term, up to ten years post-operatively [35–40]. The functional outcome assessed using single leg heel-rise test of the operated side was 40.4% of the uninjured limb, also reflecting a functional endurance deficit of the gastrosoleo complex. Given the instability sensation of most of the affected ankles, patients hesitated in performing such functional test preoperatively, and thus, only the post-operative data were available. The ATRA decreased at two-year follow-up. The ATRA assesses the passive tension of the AT at the ankle and is an indirect measure of tendon elongation: 1 cm of elongation produces an increase of dorsiflexion of 10° [22]. Following AT rupture, plantar flexion strength reduces up to 20%, and reduced plantar flexion strength may relate to greater AT lengthening [41–44].

Evidences concerning the surgical management for AT rupture which heal in continuity but results in tendon elongation are limited. For these ailments, Z-shortening has been advocated [24]. In a clinical study involving nine patients, Z-shortening restored tendon length at 32-month follow-up. The average ATRS was 83.8, demonstrating acceptable outcomes. There were two infections, which were treated successfully with antibiotics.

Tendons transmit loads from muscles to bones; thus, they must be capable of resisting high tensile forces with minimal energy loss and elongation [45]. Elongation of the AT results in reduction of plantar flexion strength, power and endurance with a subsequent reduction in activity and unsteadiness of gait. Elongation causes an unfavourable transfer of force from the gastrocnemius-soleus complex to the hindfoot, and even intensive rehabilitation cannot obviate to it. Clinically, a hypotrophic and weak calf muscle complex can be observed in most patients, with reduced ability to stand on tip toes and increased passive dorsiflexion of the foot compared to the contralateral side. Physiotherapy must be rigorously planned and systematically supervised by an expert. We advocate early weight bearing and mobilization of the ankle with progressive resistance exercises post-operatively [32, 46–48].

We employed the technique used in the present study when managing patients with chronic AT ruptures with large gaps. We previously published a report of 28 consecutive patients who underwent the procedure for chronic midportion AT rupture with a large gap (> 6 cm), reporting comparable results to the present investigation [29]. At the 2-year follow-up, the average ATRS scores significantly improved, with 93% (26 of 28) of patients reporting good-to-excellent outcomes and 57% (16 of 28) returning to their preinjury level of physical activity [29]. Only one patient experienced persistent pain over the distal wound, which improved after desensitization therapy [29]. The procedure was also performed in a cohort 28 patients with acute insertional AT rupture [27]. Overall, 82% (23 of 28) of patients reported good-to-excellent results. Two patients developed a superficial wound infection, and 79% (22 of 28) of patients returned to their preinjury activity level at a mean of 6.7 months [27]. There were only two cases of infection, which healed after systemic antibiotics were administered [27]. These investigations demonstrated that the use of a free semitendinosus graft in a minimally invasive fashion is safe and effective, associated with good outcomes also in other settings.

This study does not come without limitations. The relatively limited study size included represents the most important limitation of the present study. Future larger investigations should overcome this limitation. Although the patients were prospectively recruited, the lack of a control group is an important source of bias of the present investigation. However, there is a lack of surgical strategies to address such condition, and the condition reported in this study is uncommon. Future studies are required to investigate prognostic factors for tendon elongation in the management of AT tears to guide the decision-making process, exploring features of the mixed modalities and individual patient characteristics which may predispose to tendon elongation, and identifying the suitable candidate who can benefit at best from non-surgical strategy. Our selection and recruitment process, our assessment criteria and our follow-up were extremely rigorous and performed in strict scientific fashion. All patients were operated by a fully trained orthopaedic surgeon with a special interest in AT ailments. All the surgical procedures were performed in the same fashion and with the same instruments, modalities and materials, as was the post-operative management.

## Conclusion

The check-rein technique for AT elongation following conservative management of AT ruptures is effective and feasible to restore tendon length and calf function. The surgical outcome was influenced by the preoperative performance status, and longer time elapsed from injury to surgery worsens the outcomes. Future studies are required to investigate prognostic factors for tendon elongation to guide the decision-making process, exploring features of the therapies and individualities, which may predispose to tendon elongation, and indicating the suitable candidate who can best benefit from non-surgical management in the acute phase.

## Data Availability

The datasets generated during and/or analysed during the current study are available throughout the manuscript.

## Abbreviations

AT: Achilles tendon
ATRA: Achilles tendon resting angle
ATRS: Achilles tendon rupture score
POD: Post-operative day
ACL: Anterior cruciate ligament
